# Assessment of Ultrasonic Stress on Survival and β-Glucosidase Activity of Encapsulated *Lactiplantibacillus plantarum* BCRC 10357 in Fermentation of Black Soymilk

**DOI:** 10.3390/foods11091234

**Published:** 2022-04-25

**Authors:** Hung-Chih Tseng, Chun-Yao Yang

**Affiliations:** Department of Food Science, Fu Jen Catholic University, No. 510, Zhongzheng Rd., Xinzhuang District, New Taipei City 242062, Taiwan; jjaacckk31104@gmail.com

**Keywords:** stress response, ultrasonic stimulation, *Lactiplantibacillus plantarum*, encapsulation, fermentation

## Abstract

The enhanced β-glucosidase activity of encapsulated *Lactiplantibacillus plantarum* BCRC 10357 within calcium alginate capsules was investigated by ultrasonic stimulation to induce the stress response of the bacteria for the biotransformation of isoflavones in black soymilk. The effects of various ultrasound durations, sodium alginate concentrations (% ALG), and cell suspensions on the β-glucosidase activity of encapsulated bacteria were explored. The β-glucosidase activity of encapsulated *L. plantarum* BCRC 10357 with ultrasonic stimulation (40 kHz/300 W) was greater than that without ultrasound. With 20 min of ultrasonic treatment, the β-glucosidase activity of encapsulated *L. plantarum* BCRC 10357 from 2% ALG/0.85% NaCl cell suspension was 11.47 U/mL at 12 h, then increased to 27.43 U/mL at 36 h and to 26.25 U/mL at 48 h in black soymilk at 37 °C, showing the high adaptation of encapsulated *L. plantarum* BCRC 10357 encountering ultrasonic stress to release high β-glucosidase until 48 h, at which point the ratio of isoflavone aglycones (daidzein and genistein) in total isoflavones (daidzin, genistin, daidzein, and genistein) was 98.65%, reflecting the effective biotransformation of isoflavone glycosides into aglycones by β-glucosidase. In this study, the survivability and β-glucosidase activity of encapsulated *L. plantarum* BCRC 10357 were enhanced under ultrasonic stimulation, and were favorably used in the fermentation of black soymilk.

## 1. Introduction

Lactic acid bacteria are commonly used in the fermentation of plant foods, having beneficial functions for human health, including the reduction of cholesterol, modulation of the immunity system, and prevention of cancer, and can be served as the normal intestinal fungus group in the human gastrointestinal tract to inhibit intestinal pathogens [1,2]. During industrial production steps in food systems by lactic acid bacteria, the changes of process factors such as pH, mechanical stress, temperature, and ingredients in culture medium as well as digestion conditions may influence the growth of lactic acid bacteria by altering the functional performance to adapt to the changing environment [3,4]. When lactic acid bacteria are exposed to harsh environmental conditions, such as environmental stress during the processing and physiological stress during passage through the gastrointestinal tract, the protection for the bacteria becomes relevant, and one of the methods to avoid the bacteria from cell damages is protection by encapsulating the bacteria in biocompatible materials to ensure their beneficial effects [5,6,7].

Encapsulation is a physicochemical or mechanical process to trap substances or bacteria in some materials to form particles of nanometers to millimeters in diameter [8]. A variety of encapsulating formulations and materials have been reported for protecting lactic acid bacteria against environmental stress or encapsulating bioactive compounds to avoid degradation from processing conditions [9,10], and the viability of lactic acid bacteria could be enhanced by the encapsulation [11,12]. Various types of encapsulating materials have been applied to embed lactic acid bacteria, such as biopolymers, fats, and milk proteins [6]. However, calcium alginate is one of the simple encapsulating materials to bio-protect entrapped lactic acid bacteria, due to its non-toxicity, low cost, and biocompatibility [13]. Although the alginate encapsulation can protect lactic acid bacteria to avoid the possible damages when encountering external stress, the beneficial functions performed by the bacteria may be limited, such as the reduction of available nutrients for growth and useful enzymes released for fermentation, because of the resistance of mass transport through the calcium alginate film of the capsules [14]. This situation could be improved by applying the adaptive response of the lactic acid bacteria under ultrasound stimulation [15]. Ultrasound, regarded as non-thermal green technology, has been applied to promote the efficiency of chemical reactions and unit operations, including food processing, by generating microstreaming and local hotspots in the liquid medium from the cavitation effect [16]. Strategic ultrasonic irradiation can be used to induce the stress response of bacteria to change their physiological properties [17]. However, the adaptive responses are strain-dependent with the growth behaviors and adaptation of the bacteria regulated case by case [18].

Among the lactic acid bacteria group, *Lactiplantibacillus plantarum* is a species of Gram-positive bacteria having a high resistance and adaptation to various environmental stresses [19]. *L. plantarum* has been broadly used as a nutritional supplement in the food industry, such as for the development of probiotic formulations and biopreservation technology for food safety, and applied in the pharma industry by contributing to human medicine without causing any side effects, exhibiting benefits of antagonistic activity against pathogenic and spoilage bacteria [20,21]. Kubota et al. (2008) demonstrated that resistance to organic acids, ethanol, and sodium hypochlorite for *L. plantarum* was greater in the biofilm status, suggesting the importance of controlling lactic acid bacteria biofilms in the food industry [22]. However, the encapsulation of *L. plantarum* may influence the release of enzymes that could be useful in the fermentation. Thus, the improvement in the release of enzymes by encapsulated *L. plantarum* would be beneficial. Ultrasonic stimulation could be employed to induce the stress response of encapsulated *L. plantarum* for the enhancement of β-glucosidase released in food systems, such as black soymilk [19]. Black soymilk containing valuable isoflavones can be produced from black soybean (*Glycine max* (L.) Merr.), which is rich in anthocyanin antioxidants, saponins, carotenoids, and isoflavones, having excellent physiological functions including anti-aging effects, anti-inflammatory effects, detoxification, and relieving kidney disease [23,24]. Because the isoflavone glycosides are less bioactive than their aglycones and are hardly absorbed by intestinal epithelium tissue, increasing the content of isoflavone aglycones in black soymilk products would be beneficial to human health, and the β-glucosidase released by *L. plantarum* can be applied in the biotransformation of isoflavone glycosides into their more bioactive aglycones in foods [25]. 

During the formation of the encapsulation of lactic acid bacteria, the attachment of the bacteria on surface of alginate involves the stage of binding with the present exopolysaccharide, so as to stabilize the colony from any environmental stress [5]. However, the transport limitation of nutrients from the alginate film may cause physiological changes, differential expression of proteins, and limited growth of lactic acid bacteria [26]. Concerning the adaptive responses of the encapsulated *L. plantarum* encountering external stresses, the intensity of the ultrasonic stress may affect the viability and performance of the encapsulated bacteria. Moreover, the transport resistance for the enzyme through the calcium alginate film of the capsules is related to the type and concentration of the alginate solution that would affect the extent of the conversion of sodium alginate to calcium alginate gel [27]. Therefore, the aim of this study was to investigate the effect of ultrasonic stimulation to induce the proper stress response of encapsulated *L. plantarum* BCRC 10357 for the enhancement of β-glucosidase activity in the fermentation of black soymilk. Different types of cell suspension of *L. plantarum* BCRC 10357 were used in the encapsulation of bacteria with capsules prepared from various sodium alginate concentrations using excess calcium chloride by the extrusion method. The effects of various ultrasound durations, sodium alginate concentrations, and cell suspension liquids on the β-glucosidase activity of encapsulated *L. plantarum* BCRC 10357 as well as the efficiency of the biotransformation of isoflavones were explored.

## 2. Materials and Methods

### 2.1. Materials and Lactiplantibacillus plantarum BCRC 10357 Culture

The Tainan No. 3 black soybean (*Glycine max* (L.) Merr.), which was used to prepare the black soymilk for fermentation, was purchased from Shia Ying Farmers’ Associations (Tainan County, Taiwan). The preparation procedure of black soymilk was slightly modified from that of Tsui and Yang (2021) [4]. The black soybean was washed clean and soaked in deionized water with the ratio of 1:8 (*w*/*v*) overnight. After draining the water, the black soybeans and deionized water in the same ratio were homogenized for 3 min. Then, the black soymilk was obtained by using filtration to remove okara, and was preserved at 4 °C for use. The isoflavone standards (daidzin, genistin, daidzein, and genistein), choline chloride, alginic acid sodium salt, *p*-nitrophenyl β-D-glucopyranoside (*p*-NPG), and other chemical reagents were purchased from Sigma-Aldrich (St. Louis, MO, USA). Acetonitrile and trifluoroacetic acid were purchased from Merck (Darmstadt, Germany).

Due to *L. plantarum* having a high adaptation to environmental stress and beneficial functions to human health, *L. plantarum* BCRC 10357 was selected to investigate the responses and performances by encapsulation and ultrasonic stress. *L. plantarum* BCRC 10357 (other collection no.: ATCC 8014) was obtained from the Food Industry Research and Development Institute (Hsinchu, Taiwan) and preserved at −80 °C. *L. plantarum* BCRC 10357 was activated by inoculating in sterilized de Man, Rogosa, and Sharpe (MRS) broth in the ratio of 1% (*v*/*v*) at 37 °C for 24 h two times and used in the following encapsulation.

### 2.2. Encapsulation of L. plantarum BCRC 10357 by Extrusion

The extrusion method was employed in the encapsulation of *L. plantarum* BCRC 10357 [28]. The aliquot of 4% (*v*/*v*) *L. plantarum* BCRC 10357 (8.07 log CFU/mL) in 0.85% NaCl (or 0.5 mM choline chloride) cell suspension was well mixed with 10 mL of different concentrations of sterilized sodium alginate solution (denoted as 2% ALG, 3% ALG, and 4% ALG) and the mixture was pumped at a rate of 1 rpm/min through a syringe needle to drip into the calcium chloride solution (0.2 M). The mixture was then set to stand still for 30 min to form the capsules loaded with *L. plantarum* BCRC 10357. The collected capsules were rinsed with deionized water (sterilized) to remove the residual calcium chloride and soaked in sterile water for preservation at refrigeration condition.

The determination of the released bacteria of encapsulated *L. plantarum* BCRC 10357 followed the method of Chávarri et al. (2010) with a slight modification [29]. One gram of capsules immobilizing *L. plantarum* BCRC 10357 was homogenized with 9 mL of sodium citrate (2%, *w*/*v*) for releasing the bacteria into the solution. The growth of bacteria in the solution was measured by using the pour plate method, and the dilution liquid was prepared by 10-fold serial dilution with 0.1% (*w*/*v*) sterilized peptone water. Then, one milliliter of the dilution liquid was taken by the pour plate method, and the plate was incubated at 37 °C for 48 h for the viable cell count, which was expressed as log CFU/mL.

### 2.3. Characterization for the Microstructure of Encapsulated L. plantarum BCRC 10357

The microstructure of encapsulated *L. plantarum* BCRC 10357 was analyzed by using a field emission scanning electron microscope (FE-SEM) (JEOL, JSM-7800F, Tokyo, Japan), and followed the method of Chen et al. (2005) with a modification [30]. The encapsulated bacteria samples were dipped in 2.5% glutaraldehyde overnight for fixing. The samples were washed with 5% sucrose solution three times with each time being 10 min, and then post-fixed in 1% osmium tetroxide (prepared in 5% sucrose solution) for 60 min. After that, the capsule samples were washed again with de-ionized water three times with each time being 10 min. Subsequently, the samples were dipped in ethanol series: 15%, 30%, 50%, 60%, 70%, and 75%, each for 20 min; 80%, 85%, 90%, and 95%, each for 30 min; and 99% twice, each for 30 min. The resulting samples were preserved in 99% ethanol for analysis. The samples were first critical-point dried, and then analyzed by using FE-SEM for observing the microstructures of the capsules loaded with bacteria.

Alginates contain blocks of (1,4)-linked β-D-mannuronate (M) and α-L-guluronate (G) residues [31]. The crystallinities of polyguluronate and polymannuronate units in the capsules that were formed from different quantities of sodium alginate with CaCl_2_ were analyzed using a high-resolution X-ray diffractometer (HR-XRD). The capsule samples were scanned in the 2-theta range of 5–45° with step size of 0.02°. The intensity for the crystalline portion of polyguluronate was at about 13 of 2-theta, and that for the crystalline portion of polymannuronate was at about 22 of 2-theta [32].

### 2.4. Ultrasound Treatment on the Encapsulated L. plantarum BCRC 10357

The effect of ultrasound treatment on the viability and β-glucosidase activity of encapsulated *L. plantarum* BCRC 10357, which was prepared with 2% sodium alginate, was carried out using 40 kHz/300 W of ultrasonic bath (LEO-3002S, Leo Ultrasonic Co., New Taipei City, Taiwan) in the fermentation of black soymilk, and the power density of the ultrasonic system was 0.028 W/mL. The encapsulated bacteria were first sonicated in the sterilized black soymilk for a period of time (10, 20, and 30 min) at 30 °C using the ultrasonic bath, and then incubated at 37 °C with shaking at 150 rpm for 12–48 h. The viable cell count and β-glucosidase activity were then determined.

### 2.5. Determination of β-Glucosidase Activity

The determination of β-glucosidase activity followed the method of Tsui and Yang (2021) with a slight modification [4], and was based on measuring the amount of *p*-nitrophenol released from the hydrolysis rate of *p*-NPG. One milliliter of fermentation medium was mixed with 100 μL of 5 mM *p*-NPG (prepared from 0.1 M of phosphate buffer solution at pH 7.0) to react at 37 °C for 30 min. The reaction was then terminated by adding 50 μL of 1.0 M Na_2_CO_3_. After that, the mixture was centrifuged at 12,000 rpm for 30 min, and the supernatant was taken for the analysis of β-glucosidase activity (expressed as U/mL) using a spectrophotometer at 405 nm (Hitachi, Ratio Beam Spectrophotometer U-5100, Tokyo, Japan).

### 2.6. Determination of Isoflavones

The isoflavone contents in the fermented product extract were determined by high performance liquid chromatography (HPLC) using daidzin, daidzein, genistin, and genistein (Sigma-Aldrich, St. Louis, MO, USA) as the standards for quantification. The HPLC system was equipped with a UV-VIS detector (Hitachi Chromaster 5420 UV-VIS detector, Hitachi, Tokyo, Japan) and Mightysil RP-18 column (5 μm, 250 mm × 4.6 mm, Kanto Chemical Co., Tokyo, Japan), and the conditions of HPLC followed the method of Yu and Yang (2019) with 0.1% (*v*/*v*) trifluoroacetic acid (solvent A) and acetonitrile (solvent B) as the mobile phase set at 0.8 mL/min of flow rate [33]. The gradients were set as solvent A: 90% at 0 to 10 min, 90% to 45% at 10 to 35 min, 45% to 90% at 35 to 45 min, and 90% at 45 to 60 min.

### 2.7. Statistical Analysis

Each experiment was performed in triplicate by using three independent samples, and the experimental data were expressed as mean ± standard deviation (*n* = 3). By using IBM SPSS statistics 20 (IBM Corp., Armonk, NY, USA), one-way analysis of variance (ANOVA) with Duncan’s multiple range tests was applied for the statistical analysis of data, and statistical significance was determined at *p* < 0.05.

## 3. Results and Discussion

### 3.1. Effect of Culture Medium on L. plantarum BCRC 10357

**Figure 1:**
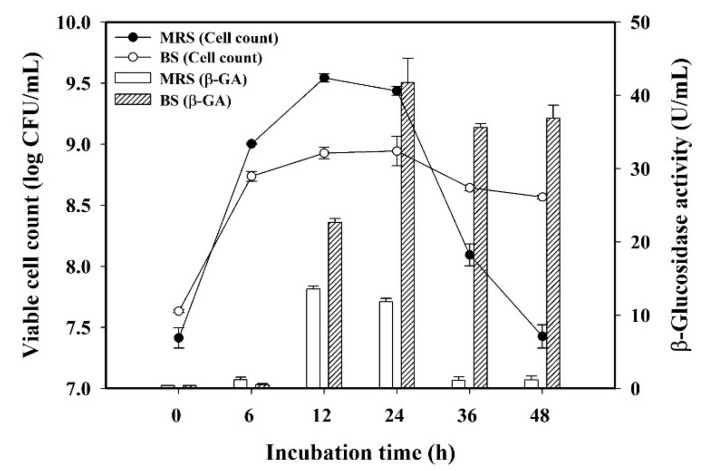
Variations of viable cell count and β-glucosidase activity (β-GA) of *L. plantarum* BCRC 10357 in MRS broth and black soymilk (BS) at 37 °C during 48 h of incubation. Data were expressed as mean ± standard deviation with triplicate experiment (*n* = 3).

To assess the adaptation behavior of *L. plantarum* BCRC 10357 by the change of the medium environment, the viable cell counts and β-glucosidase activity of *L. plantarum* BCRC 10357 incubated in black soymilk and in MRS broth at 37 °C for 48 h were analyzed, as shown in Figure 1. It was found that the viable cell count using MRS was greater than that using black soymilk before 24 h of incubation, and the maximum value (9.54 log CFU/mL) was found at 12 h of incubation using MRS broth. At 48 h of incubation, the viable cell count with MRS was quickly reduced to 7.43 log CFU/mL, but the viable cells using black soymilk could be maintained at 8.57 log CFU/mL. However, the β-glucosidase activities of the bacteria using black soymilk were all greatly higher than that using MRS broth in the period of 12 to 48 h of incubation with the maximum β-glucosidase activity of 41.74 U/mL at 24 h of incubation. It was also revealed that the release of β-glucosidase per unit cell of *L. plantarum* BCRC 10357 was higher in black soymilk than in MRS broth, and the bacteria had a proper adaptation to the change of the medium environment by regulating their physiological behavior for survival.

### 3.2. Morphological Structure and XRD Analysis of Capsules Loaded with L. plantarum BCRC 10357

**Figure 2:**
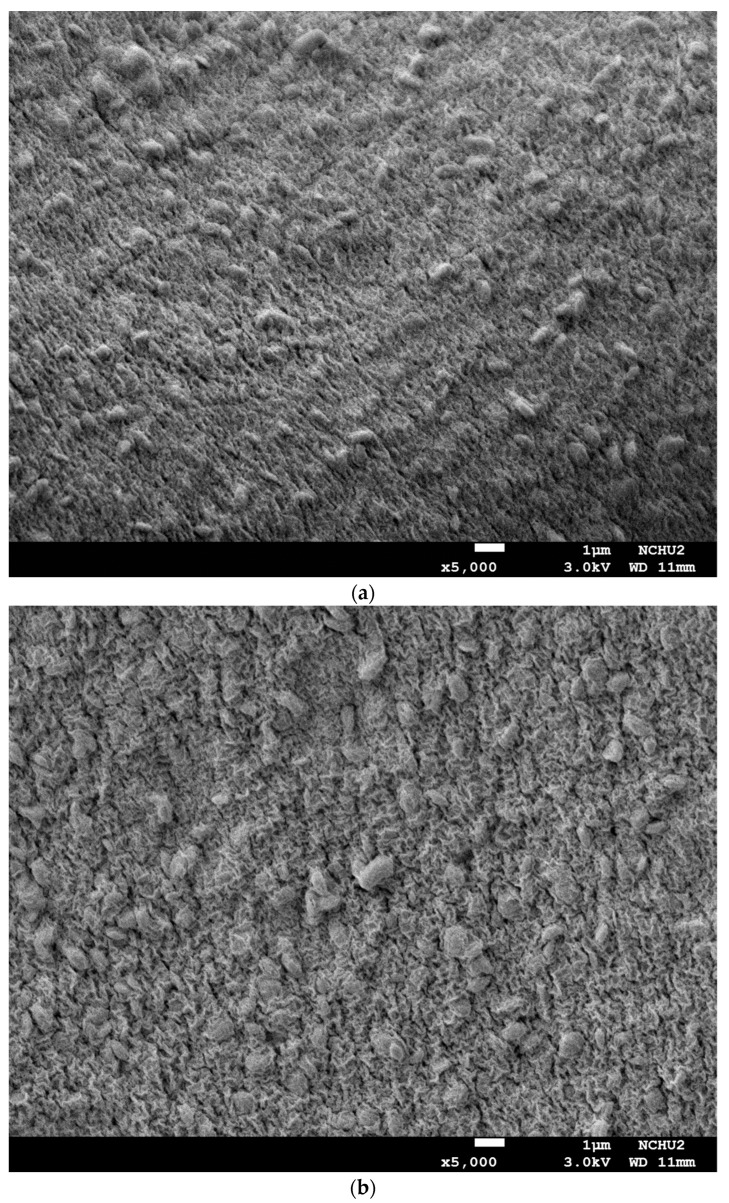

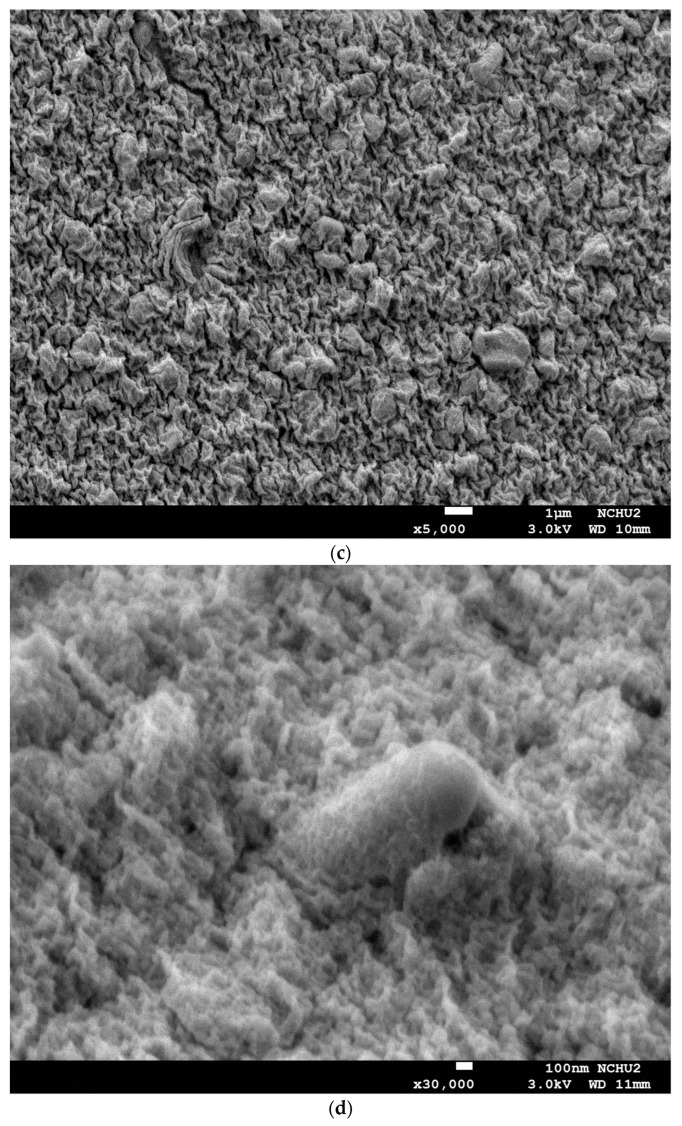

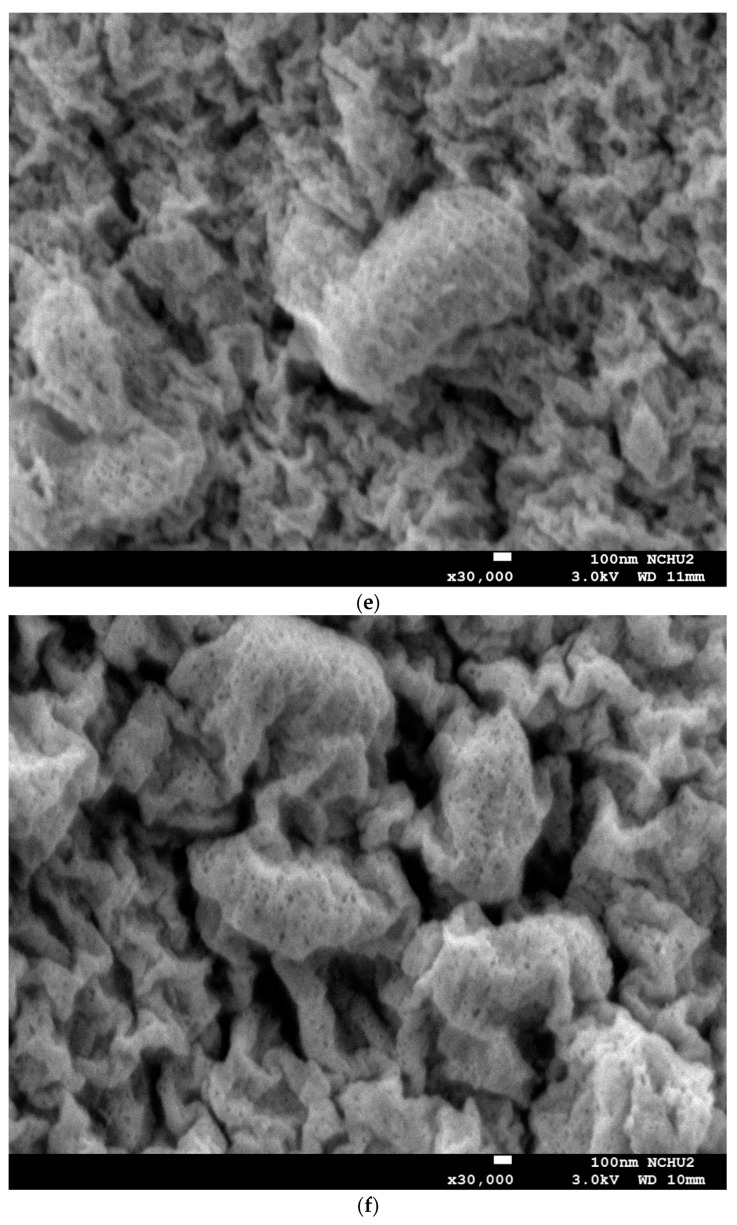
FE-SEM images of capsules loaded with *L. plantarum* BCRC 10357 by using different concentrations of sodium alginate (% ALG): (**a**) 2% ALG capsule (×5000), (**b**) 3% ALG capsule (×5000), (**c**) 4% ALG capsule (×5000), (**d**) bacteria embedded in 2% ALG capsule (×30,000), (**e**) bacteria embedded in 3% ALG capsule (×30,000), (**f**) bacteria embedded in 4% ALG capsule (×30,000).

Figure 2 shows the FE-SEM images of capsules loaded with *L. plantarum* BCRC 10357 using different concentrations of sodium alginate (% ALG) and fixed by glutaraldehyde. As is shown in Figure 2a for 2% ALG (×5000), Figure 2b for 3% ALG (×5000), and Figure 2c for 4% ALG (×5000), the surface of the capsules for 4% ALG was much tighter in the gel wrinkle structure than that for 2% and 3% ALG, and the bacteria were observed to be effectively embedded in the surface of the capsules. Furthermore, the variations for the bacteria encapsulated in the capsule surfaces with different concentrations of sodium alginate were observed in Figure 2d–f for 2% ALG (×30,000), 3% ALG (×30,000), and 4% ALG (×30,000), respectively. The gel structure of the surface of 2% ALG capsules was much smoother with fewer wrinkles than that of 3% and 4% ALG capsules, and the bacteria were much more distinctly observed to be embedded in the surface of the capsules. This possibly resulted from the gelling of alginate with calcium ion starting from the cell wall of the bacteria before the arrival of external calcium ion, especially for a higher concentration of sodium alginate [34].

**Figure 3:**
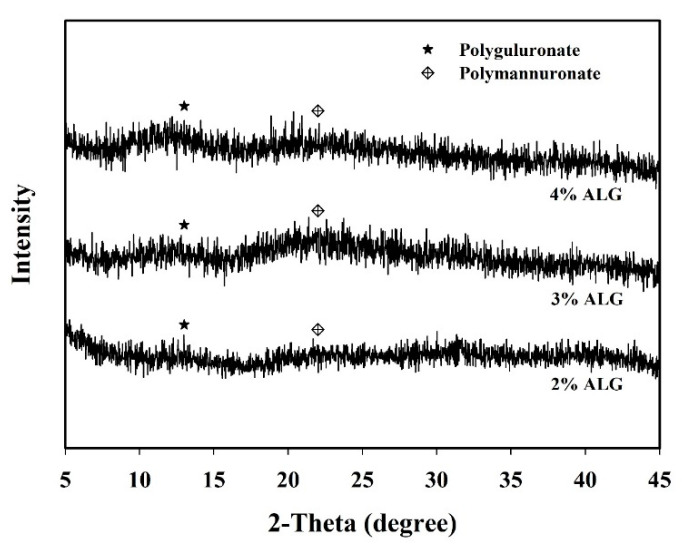
XRD patterns for the capsules loaded with *L. plantarum* BCRC 10357 by using different concentrations of sodium alginate (% ALG).

The microstructure of alginate capsules depends on several factors, including internal molecular arrangements, the concentrations of guluronate and mannuronate residues, and interaction between alginate and polycation [35]. Figure 3 shows the HR-XRD analysis of the alginate capsules loaded with *L. plantarum* BCRC 10357 prepared from different concentrations of sodium alginate. The peaks at 2θ = 13 and 22 depicted the crystalline portions of polyguluronate and polymannuronate units, respectively [32,36]. The intensity of polyguluronate crystalline portions in alginate increased with increasing the concentration of sodium alginate used, while the intensities of the polymannuronate crystalline portions in alginate for 2% ALG and 4% ALG capsules were slightly less intensified than that for 3% ALG capsules. This might be due to the amounts of guluronic acid and mannuronic acid present in the different alginate samples [37,38].

### 3.3. Stress Response of Encapsulated L. plantarum BCRC 10357 by Ultrasonic Treatment

**Figure 4:**
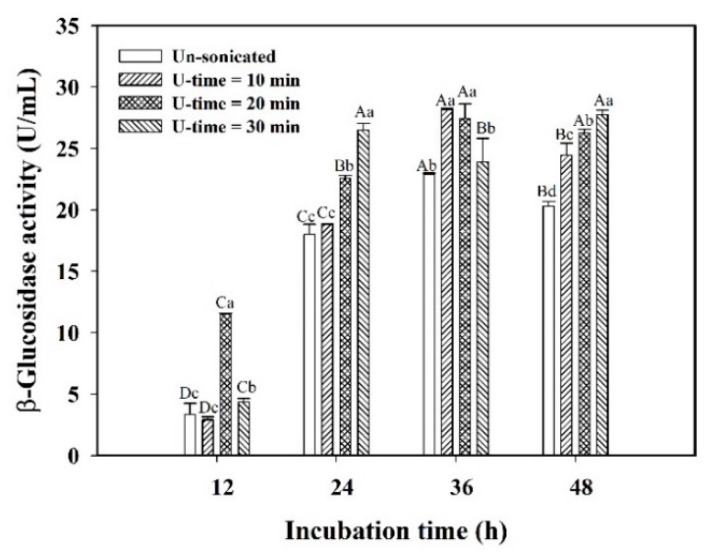
Effect of different durations of ultrasonic treatment (40 kHz/300 W) (U-time) on the β-glucosidase activity of encapsulated *L. plantarum* BCRC 10357 within 2% ALG capsules in black soymilk at 37 °C. Data were expressed as mean ± standard deviation with triplicate experiment (*n* = 3). Values with different lowercase letters at the same incubation time and different uppercase letters at the same U-time were significantly different (*p* < 0.05) by Duncan’s multiple range test.

By the protection of capsule walls for encapsulated *L. plantarum* BCRC 10357 against environmental stress, the response of the bacteria encountering ultrasonic stimulation might induce β-glucosidase to release to regulate their growth and adapt to the new environment for survival. Various durations of ultrasonic stimulation (40 kHz/300 W) (U-time) on the encapsulated *L. plantarum* BCRC 10357 in 2% ALG capsules were performed. The viable cell counts and β-glucosidase activities of the encapsulated bacteria during 48 h of incubation in black soymilk are shown in Table 1 and Figure 4, respectively. The viable cell counts of the encapsulated bacteria were not significantly different between the cases without or with various durations of ultrasonic treatment (Table 1). The viable cell counts reached the highest values at 36 h of incubation, and the viability of the encapsulated bacteria would be kept at a high level until 48 h of incubation with the cell count in the range of 8.73–8.82 log CFU/mL. This showed that the capsule walls could effectively provide the shielding to protect the encapsulated bacteria from the possible cell damages caused by the cavitation effect of ultrasound irradiation, which would generate microstreaming and local hotspots in the liquid medium. Although the viable cell counts of the encapsulated bacteria were insignificantly influenced by ultrasonic treatment, the ultrasonic stimulation would still induce stress the response of encapsulated *L. plantarum* BCRC 10357 by regulating physiological behaviors in releasing β-glucosidase to adapt to environmental changes from various durations of ultrasonic stimulation, due to which the binding of the bacteria to the alginate surface with exopolysaccharide would change the physiological behavior and express different responses to environmental stresses [5,26].

The effects of ultrasonic stimulation and incubation time on the β-glucosidase activity of encapsulated *L. plantarum* BCRC 10357 were evaluated. As is shown in Figure 4, the β-glucosidase activities of encapsulated *L. plantarum* BCRC 10357 with various durations of ultrasonic stimulation were all greater than that without ultrasonic treatment. Without ultrasonic stimulation, the β-glucosidase activity of encapsulated *L. plantarum* BCRC 10357 merely increased from 3.33 U/mL at 12 h of incubation to 22.89 U/mL at 36 h, then significantly decreased to 20.30 U/mL at 48 h of incubation; while with 20 min of ultrasonic treatment, the β-glucosidase activity was 11.47 U/mL at 12 h, and increased significantly to 27.43 U/mL at 36 h, then reduced insignificantly to 26.25 U/mL at 48 h of incubation, indicating that the β-glucosidase activity would be kept at a more stable and high value until 48 h via 20 min of ultrasonic treatment (Figure 4). Such enhancement in β-glucosidase activity resulted from the response of the encapsulated bacteria on ultrasonic stimulation by regulating physiological behavior to release more β-glucosidase translocating across the cytoplasmic membrane to extracellular sites and the medium [15,39,40]. However, the β-glucosidase activity of the encapsulated bacteria with 30 min of U-time was less than that with 20 min of U-time at 12 h of incubation, but higher than that with 20 min of U-time at 24 h of incubation. This exhibited that the encapsulated bacteria under 30 min of ultrasonic stimulation needed a longer time to regulate the physiological behavior for survival. This also demonstrated that a proper level of ultrasonic stimulation on encapsulated *L. plantarum* BCRC 10357 would be better to induce the favorable response of the encapsulated bacteria for performing the beneficial functions [17].

### 3.4. Effect of Encapsulation Modes on L. plantarum BCRC 10357 in the Fermentation of Black Soymilk

**Figure 5:**
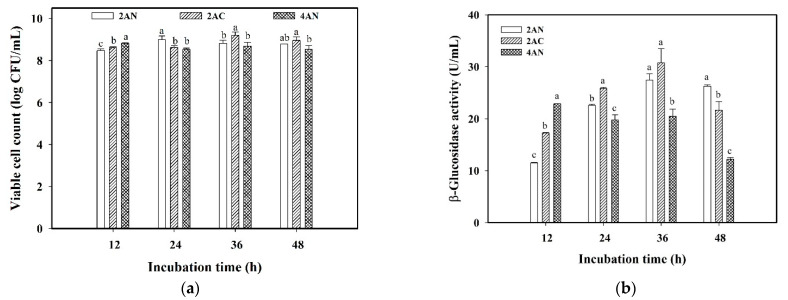
Variations of different encapsulation modes on (**a**) viable cell count and (**b**) β-glucosidase activity of encapsulated *L. plantarum* BCRC10357 in black soymilk at 37 °C. Encapsulation modes: 2% ALG/0.85% NaCl suspension (2AN), 2% ALG/0.5 mM choline chloride suspension (2AC), and 4% ALG/0.85% NaCl suspension (4AN). Data were expressed as mean ± standard deviation (*n* = 3). Different superscript letters at the same incubation time were significantly different (*p* < 0.05) by Duncan’s multiple range test.

The cell suspension liquid and sodium alginate concentration may affect the viability of *L. plantarum* BCRC 10357 and the release of β-glucosidase in the medium. Three encapsulation modes of 2% ALG/0.85% NaCl suspension (denoted as 2AN), 2% ALG/0.5 mM choline chloride suspension (denoted as 2AC), and 4% ALG/0.85% NaCl suspension (denoted as 4AN) were compared for the encapsulation of *L. plantarum* BCRC 10357 that would be used in the fermentation of black soymilk at 37 °C via 20 min of ultrasonic stimulation (40 kHz/300 W). The viable cell count and β-glucosidase activity for the encapsulation modes of 2AN, 2AC, and 4AN are shown in Figure 5a,b, respectively. In 12 h of incubation, the viable cell counts showed the order of 4AN (8.82 log CFU/mL) > 2AC (8.62 log CFU/mL) > 2AN (8.47 log CFU/mL) significantly (Figure 5a). This order of greater or lesser viable cell count was consistent with that of β-glucosidase activity at 12 h of incubation, i.e., 4AN (22.857 U/mL) > 2AC (17.287 U/mL) > 2AN (11.473 U/mL) (Figure 5b). Furthermore, the mode 2AC showed the highest β-glucosidase activity (30.760 U/mL) at 36 h of incubation, but having an insignificant difference from 2AN (27.433 U/mL); while in a longer fermentation time of 48 h, the order of β-glucosidase activity changed significantly as 2AN (26.250 U/mL) > 2AC (21.647 U/mL) > 4AN (12.200 U/mL) (Figure 5b). It was speculated that the transport resistance for β-glucosidase released by the encapsulated *L. plantarum* BCRC 10357 with 4% ALG was more significant in a longer fermentation time, due to the more wrinkled structure in 4% ALG capsules and the adaptive response of the encapsulated bacteria [14]. It demonstrated that *L. plantarum* BCRC 10357 encapsulated from 2% ALG combined with 0.85% NaCl suspension was able to properly respond to the environmental stress of 20 min ultrasound irradiation for survival, and maintained a high β-glucosidase activity at 48 h of incubation. It also showed that, compared with 0.85% NaCl suspension, 0.5 mM choline chloride as the cell suspension could provide different nutritional effects on the bacteria for the adaptation to the ultrasonic stress [26]. From Figure 5a, although the viable cell counts did not differ much for the various encapsulation modes, the β-glucosidase activities of encapsulated *L. plantarum* BCRC 10357 showed significant differences for different encapsulation modes (Figure 5b). This revealed that the adaptation behavior of *L. plantarum* BCRC 10357 was quite affected by the type of encapsulation mode, resulting in different performances of the bacteria in releasing β-glucosidase.

The high β-glucosidase activity released by *L. plantarum* BCRC 10357 was employed to bio-transform the isoflavone glycosides into their aglycones in the fermentation of black soymilk. Table 2 shows the comparison of the contents of isoflavone glycosides (daidzin and genistin) and their aglycones (daidzein and genistein) in the dried black soymilk between 12 h (early time) and 48 h (late time) of fermentation by using encapsulated *L. plantarum* with 20 min of ultrasonic stimulation. The ratio of isoflavone aglycones (daidzein and genistein, AI) in the total isoflavones (daidzin, genistin, daidzein, and genistein, TI) reflected the level of the biotransformation of isoflavone glycosides into their aglycones by β-glucosidase. As shown in Table 2, the orders of the ratio AI/TI were 4AN (72.34%) > 2AC (59.56%) > 2AN (18.65%) in 12 h of fermentation and 2AN (98.65%) > 2AC (86.46%) > 4AN (84.09%) in 48 h of fermentation, showing that the order of the biotransformation level of isoflavones corresponded to the order of the β-glucosidase activity of the encapsulated bacteria after 20 min of ultrasonic treatment before fermentation (Figure 5b). Therefore, using the cell suspension of 0.85% NaCl could cause the encapsulated *L. plantarum* BCRC 10357 to release the more stable β-glucosidase activity for the fermentation of black soymilk than using 0.5 mM choline chloride as the cell suspension. Under ultrasonic stimulation, the best performance of encapsulated *L. plantarum* BCRC 10357 was that prepared from 2% ALG and 0.85% NaCl cell suspension.

## 4. Conclusions

In this study, the effect of ultrasonic stimulation to induce the proper stress response for the enhancement in the β-glucosidase activity of encapsulated *L. plantarum* BCRC 10357 for the fermentation of black soymilk was investigated. The capsules were prepared from various sodium alginate concentrations with excess calcium chloride by the extrusion method. The β-glucosidase activity of encapsulated *L. plantarum* BCRC 10357 with various durations of ultrasonic stimulation (40 kHz/300 W) was all greater than that without ultrasound, and the duration of 20 min of ultrasonic stimulation was the best to induce the proper response of encapsulated bacteria by releasing the high β-glucosidase. Moreover, the use of 0.85% NaCl as the cell suspension liquid resulted in a more stable and higher β-glucosidase activity than that using 0.5 mM choline chloride as the cell suspension in 48 h of incubation. In summary, the high adaptation of encapsulated *L. plantarum* BCRC 10357 encountering the environmental stress of ultrasound irradiation was achieved from regulating the physiological behaviors to release high β-glucosidase for survival, by which the high level of the biotransformation of isoflavones glycosides to their aglycones in black soymilk was attained, giving the ratio of 98.65% of isoflavone aglycones in total isoflavones. In this study, the methodology of ultrasonic stimulation to enhance the β-glucosidase activity of encapsulated *L. plantarum* BCRC 10357 was developed in the fermentation of black soymilk used as a functional food, and has the potential to be applied in the fermentation of food systems.

## Figures and Tables

**Table 1:** Effect of different durations of ultrasonic treatment (40 kHz/300 W) (U-time) on viable cell counts of encapsulated *L. plantarum* BCRC10357.

U-Time (Min)	Viable Cell Count (Log CFU/mL) at Incubation Time (h) *			
	12	24	36	48
Un-sonicated	8.45 ± 0.03 ^a^	8.70 ± 0.02 ^b^	8.90 ± 0.15 ^a^	8.73 ± 0.03 ^b^
10	8.28 ± 0.14 ^a^	8.80 ± 0.10 ^ab^	8.81 ± 0.27 ^a^	8.77 ± 0.08 ^ab^
20	8.47 ± 0.09 ^a^	8.99 ± 0.17 ^a^	8.81 ± 0.15 ^a^	8.78 ± 0.02 ^ab^
30	8.49 ± 0.18 ^a^	8.79 ± 0.05 ^b^	8.83 ± 0.14 ^a^	8.82 ± 0.01 ^a^

* Conditions: 2% ALG capsules in black soymilk, 37 °C. U-time means the duration of ultrasonic treatment (40 kHz/300 W), and ‘Un-sonicated’ means U-time = 0 min. Data were expressed as the mean ± standard deviation with a triplicate experiment (*n* = 3). Different superscript letters at the same incubation time were significantly different (*p* < 0.05) by Duncan’s multiple range test.

**Table 2:** Effect of encapsulation modes of encapsulated *L. plantarum* BCRC 10357 on the biotransformation of isoflavones in black soymilk at 37 °C *.

Items	Fermentation Time (h)	Isoflavones (μg/g—Dried Black Soymilk)				AI/TI (%)
		Daidzin	Genistin	Daidzein	Genistein	
2AN	12	132.67 ± 20.24	166.11 ± 17.64	46.09 ± 9.15	22.22 ± 3.65	18.65
2AC	12	83.89 ± 32.87	63.35 ± 24.70	143.25 ± 30.24	65.36 ± 15.73	59.56
4AN	12	70.17 ± 15.37	11.43 ± 1.67	151.01 ± 34.50	64.07 ± 18.60	72.34
2AN	48	2.36 ± 0.19	2.44 ± 0.07	195.40 ± 30.67	162.95 ± 28.16	98.65
2AC	48	14.50 ± 7.29	28.69 ± 15.87	161.68 ± 68.47	101.48 ± 47.47	86.46
4AN	48	18.33 ± 5.04	39.93 ± 12.14	193.14 ± 31.42	109.26 ± 17.70	84.09

* The encapsulated *L. plantarum* BCRC 10357 was treated with ultrasound (40 kHz/300 W) for 20 min. Data were expressed as mean ± standard deviation (*n* = 3). Encapsulation modes: 2% ALG/0.85% NaCl suspension (2AN), 2% ALG/0.5 mM choline chloride suspension (2AC), and 4% ALG/0.85% NaCl suspension (4AN). AI = daidzein + genistein, TI = daidzin + genistin + daidzein + genistein.

## Data Availability

Data are contained within the article.
